# Prognostic utility of systemic inflammatory markers and chronic hepatitis C virus infection status in hepatocellular carcinoma patients treated with local ablation

**DOI:** 10.1186/s12885-021-09121-8

**Published:** 2022-02-28

**Authors:** Mohamed Abdulwahab Mohamed Ali, William Scott Harmsen, Khairy Hammam Morsy, Ghada Moustapha Kamal Galal, Terry M. Therneau, Lewis Rowland Roberts

**Affiliations:** 1 Department of Tropical Medicine and Gastroenterology, Sohag Faculty of Medicine, Naser City, Sohag, 82524 Egypt; 2 Division of Clinical Trials and Biostatistics, Department of Quantitative Health Sciences, Mayo Clinic College of Medicine and Science, 205 Third Street SW, Rochester, MN 55905 USA; 3 Division of Gastroenterology and Hepatology, Mayo Clinic College of Medicine and Science, 200 First Street SW, Rochester, MN 55905 USA

**Keywords:** Hepatocellular carcinoma, Radiofrequency ablation, Microwave ablation, Hepatitis C virus, Clinical outcomes, Lymphocyte monocyte ratio

## Abstract

**Background:**

Hepatocellular carcinoma (HCC) has high incidence and mortality worldwide. Local ablation using radiofrequency ablation (RFA) or microwave ablation (MWA) is potentially curative for early-stage HCC with outcomes comparable to surgical resection. We explored the influence of demographic, clinical, and laboratory factors on outcomes of HCC patients receiving ablation.

**Methods:**

This retrospective cohort study included 221 HCC patients receiving local ablation at Mayo Clinic between January 2000 and October 2018, comprising 140 RFA and 81 MWA. Prognostic factors determining overall survival (OS) and disease-free survival (DFS) were identified using multivariate analysis.

**Results:**

There was no clinically significant difference in OS or DFS between RFA and MWA. In multivariate analysis of OS, pre-ablation lymphocyte-monocyte ratio [Hazard ratio (HR) 0.7, 95% confidence interval (CI) 0.58–0.84, *P* = 0.0001], MELD score [HR 1.12, 95%CI 1.068–1.17, *P* <  0.0001], tumor number [HR 1.23, 95%CI 1.041–1.46, *P* = 0.015] and tumor size [HR 1.18, 95%CI 1.015–1.37, *P* = 0.031] were clinically-significant prognostic factors. Among HCC patients with chronic hepatitis C (HCV) infection, positive HCV PCR at HCC diagnosis was associated with 1.4-fold higher hazard of death, with 5-year survival of 32.8% vs 53.6% in HCV PCR-negative patients. Regarding DFS, pre-ablation lymphocyte-monocyte ratio [HR 0.77, 95%CI 0.66–0.9, *P* = 0.001], MELD score [HR 1.06, 95%CI 1.022–1.11, *P* = 0.002], Log^2^ AFP [HR 1.11, 95%CI 1.033–1.2, *P* = 0.005], tumor number [HR 1.29, 95%CI 1.078–1.53, *P* = 0.005] and tumor size [HR 1.25, 95%CI 1.043–1.51 *P* = 0.016] were independently prognostic.

**Conclusions:**

Pre-ablation systemic inflammation represented by lymphocyte-monocyte ratio is significantly associated with OS and DFS in HCC patients treated with local ablation. HCV viremia is associated with poor OS. Tumor biology represented by tumor number and size are strongly prognostic for OS and DFS while AFP is significantly associated with DFS only.

## Background

Hepatocellular carcinoma (HCC) is the sixth most common cancer and the fourth most common cause of cancer-associated death worldwide in 2018, with 841,000 new cases and 782,000 deaths annually. Incidence and mortality rates are 2–3 times higher in men than in women. Consequently, HCC is the fifth most common cancer and the second most common cause of cancer death in men [1]. Patients diagnosed with very early or early-stage tumors are potentially curable with surgical resection, liver transplantation or local ablation, achieving 5-year survival rates ranging from 40 to 70% [2]. However, most patients are diagnosed with intermediate or advanced stage disease and have limited survival due to the poor effectiveness of the available treatment options and the progression of coexistent chronic liver disease.

Chemical local ablation using percutaneous ethanol injection (PEI) was first described in 1983 and was shown to be safe, simple and affordable [3]. However, PEI has been largely superseded by the thermal therapies of radiofrequency ablation (RFA) and microwave ablation (MWA), which have been proven to be safer, more convenient and more effective in producing tumor necrosis and preventing tumor recurrence [4]. Additionally, there are other newer ablation modalities such as laser ablation, cryoablation, irreversible electroporation and high-intensity–focused ultrasound ablation that are under evaluation and have shown utility in specific circumstances [5–8]. The emergence of these efficient ablation techniques and their incorporation into evidence-based treatment guidelines has established the clinical utility of local ablation as a curative treatment option for early stage HCC.

Local ablation is used not only for treatment of small HCCs with curative intent but also as a bridging therapy prior to liver transplantation for HCC [6]. Given the scarcity of resources available for liver transplantation, with a limited supply of donors and specialized centers with highly trained surgeons, it is important to explore the possible prognostic factors that may influence patient outcomes after pre-transplant local ablation of HCCs.

One third of all HCCs globally are attributed to chronic hepatitis C virus (HCV), with a 1–8% annual risk in patients with HCV-induced cirrhosis [9]. End-stage liver failure is the primary cause of death in HCV-cirrhotic patients after curative treatment of HCC, highlighting the importance of maintaining hepatic function in these patients [10]. Management of HCV has been transformed over the past few years with the emergence of direct acting antiviral drugs (DAA). However, the majority of studies have focused on HCC development following DAA therapy compared to previous IFN-based regimens. Relatively few studies have examined the impact of co-management of active HCV and HCC [11].

There is growing evidence that chronic inflammation promotes oncogenesis and inflammation has been linked to poor prognosis in cancer patients. The nature and magnitude of inflammation was found to be negatively correlated with survival of cancer patients [12]; a higher neutrophil count is linked to an impaired anti-tumor immune response, augmented synthesis and systemic release of vascular endothelial growth factor (VEGF) [13] which plays a pivotal role in tumor angiogenesis and hence higher tumor recurrence rates [14]. Lymphopenia is an integral component of this inflammatory reaction; suppression of the antitumor T-lymphocyte response with generation of dysfunctional cytotoxic CD81 lymphocytes has been demonstrated in many studies [15]. Lastly, circulating monocytes have been reported to enhance tumor growth and potentiate immune escape of tumor cells [16] and their infiltration into HCC has been linked to a more aggressive behavior of the tumor [17]. Consequently, the neutrophil to lymphocyte ratio (NLR), lymphocyte to monocyte ratio (LMR) and platelet to lymphocyte ratio (PLR) have been investigated as potential prognostic tools for HCC patients; particularly those receiving surgical resection [18], transplantation [19] and systemic treatment with sorafenib [20]. Much less is known about the prognostic roles of these ratios in patients treated by local ablation.

In clinical practice we have observed that many HCC patients with apparently similar baseline characteristics have diverse clinical outcomes although they received the same treatments, including local ablation. Therefore, we proposed this study to better understand the determinants of outcome of HCC patients receiving local ablation therapy in order to develop predictors of the outcome. We examined systemic inflammatory markers (NLR, LMR and PLR) and underlying liver disease (chronic HCV was the most prevalent etiology) as key predictors of patients’ prognosis. Additionally, well-known predictors of outcome such as tumor characteristics (size and number) and alpha-fetoprotein (AFP) were evaluated to provide a more comprehensive assessment of this population, with the ultimate goal of enhancing treatment selection and patient prognostication.

## Methods

### Patients

This study was approved by the Institutional Review Board at Mayo Clinic Rochester USA. Informed consent was waived by the same committee due to the minimal risk nature of the study, which involved only chart review with no patient contact. All study related activities were performed in accordance with the Declaration of Helsinki. HCC patients who underwent local ablation as their primary therapy, including 12 patients who underwent ablation as a bridge to liver transplantation, between January 2000 and October 2018 at Mayo Clinic Rochester were included. Patients with recurrent HCC, ablation combined with other treatments e.g., embolic therapy, uncommon ablation techniques or those with hepatic metastasis from other primary sites were excluded.

The diagnosis of HCC was based on a combination of radiological and pathological criteria according to the generally accepted guidelines for HCC diagnosis at the time of initial diagnosis.

Variables abstracted from the electronic medical record included demographics (age, sex and gender), presence of cirrhosis and underlying liver disease, portal hypertension, Child-Turcotte-Pugh (CTP) score, Model for End-Stage Liver Disease (MELD) score, and Barcelona Clinic Liver Cancer (BCLC) class at initial presentation, laboratory tests performed prior to local ablation (CBC, AFP, thyroid hormones, cholesterol), and tumor characteristics (tumor size and number).

### Definitions of NLR, LMR and PLR

Pre-ablative white blood cell count and differential counts obtained within 1 month before tumor ablation were abstracted. The pre-procedure NLR was calculated from the differential count by dividing the absolute neutrophil count by the absolute lymphocyte count. The LMR was calculated by dividing the absolute lymphocyte count by the absolute monocyte count and the PLR was calculated by dividing the absolute platelet count by the absolute lymphocyte count. The post-procedure NLR was obtained at the first follow-up visit at the outpatient department 1 to 3 months after ablation.

### Follow-up and outcomes

All patients were followed up every 3–6 months during the first 2 years and approximately every 6 months subsequently until death or September 1st, 2020. Follow up evaluation included liver tests, AFP and imaging with multiphasic CT or dynamic MRI. The primary outcomes were overall survival (OS) and disease-free survival (DFS). DFS was defined as a combination of death and/or tumor recurrence (the time period from ablation to identification of tumor relapse on imaging).

### Statistical analysis

Continuous variables were reported as mean ± standard deviation. Differences in categorical variables and continuous variables between the groups were analyzed with the chi square test or Fisher’s exact test and with Student’s *t* test, respectively. OS and DFS curves were evaluated using Kaplan–Meier curves and compared using the log-rank test. Variables of interest in the univariate analysis were entered into a Cox proportional hazards model for multivariate analysis. As there were a sufficient number of events all non-correlated variables were retained in the multivariable model. To deal with the problem of multicollinearity, we removed variables that are highly correlated from the multivariate analysis. For the variables of interest that were overlapping, including the neutrophil to lymphocyte ratio (NLR) and lymphocyte to monocyte ratio (LMR), we ran multiple models to test these dependent variables separately. Two-tailed *P* values less than 0.05 were considered statistically significant. All statistical methods were implemented in SAS statistical software (SAS Institute, Cary, NC, USA), version 9.4.

## Results

### Baseline characteristics

**Fig. 1:**
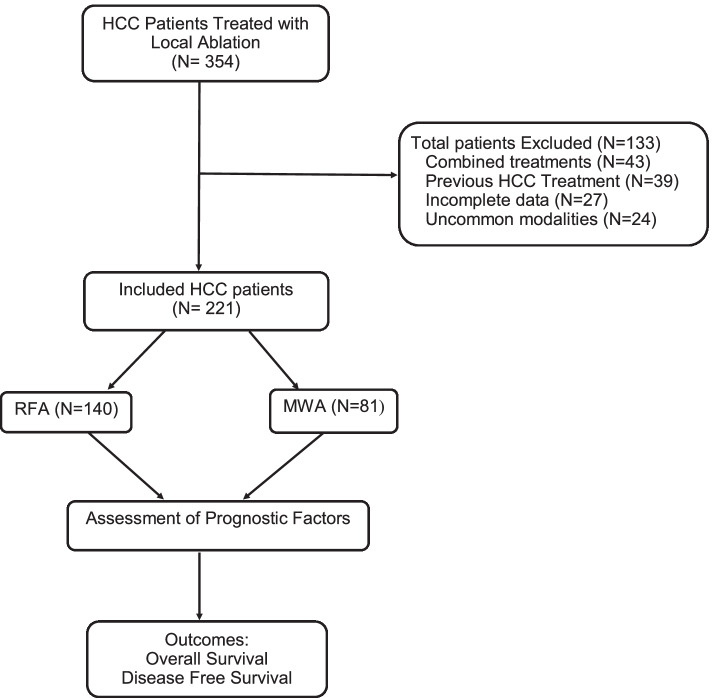
Study flow chart

Three hundred fifty-four patients with HCC who underwent local ablation were identified, of these 133 patients were excluded due to simultaneous use of combined treatment modalities e.g., ablation with embolic therapy or two local ablation techniques (43 patients), previous HCC treatment (39 patients), missing laboratory or imaging data (27 patients), and use of uncommon ablation techniques such as laser ablation or cryoablation (24 patients) (Fig. 1). The remaining 221 HCC patients who underwent local ablation as primary therapy were included in the study; 140 (63.3%) received RFA and 81 (36.7%) received MWA. Within the entire cohort 12 patients (5.4%) subsequently received liver transplantation, thus local ablation of their tumors was considered a bridging therapy and these patients were censored at the time of transplantation.

The study population included 142 males (64.3%) and 79 females (35.7%), with a mean age at ablation procedure of 67.9 ± 9.6 years (range 33.3 to 88.4). The median overall follow-up time was 69.6 months. The baseline characteristics of patients in the two groups are shown in Table 1**.** There were no significant differences between the groups in the baseline characteristics for most variables e.g., age, gender, underlying liver disease, cirrhosis, portal hypertension, AFP or inflammatory markers.

**Table 1:** Baseline Characteristics for the ablation groups

	MWA (*N* = 81)	RFA (*N* = 140)	Total (*N* = 221)	*P*-value
**Age, years**				0.8^1^
Mean ± SD	68.5 ± 8.1	67.6 ± 10.4	67.9 ± 9.6	
**Gender, no. (%)**				0.082^2^
Female	23 (28.4%)	56 (40%)	79 (35.7%)	
Male	58 (71.6%)	84 (60%)	142 (64.3%)	
**Cirrhosis, no. (%)**				0.52^2^
No	5 (6.2%)	12 (8.6%)	17 (7.7%)	
Yes	76 (93.8%)	128 (91.4%)	204 (92.3%)	
**Underlying Liver Disease, no. (%)**				0.14^2^
Hepatitis C virus	27 (33.3%)	56 (40%)	83 (37.6%)	
Non-alcoholic fatty liver	26 (32.1%)	25 (17.9%)	51 (32.1%)	
Alcoholic Liver disease	12 (14.8%)	22 (15.7%)	34 (15.4%)	
Hepatitis B virus	5 (6.2%)	7 (5%)	12 (5.4%)	
Others	11 (13.6%)	30 (21.4%)	41 (18.6%)	
**Portal Hypertension, no. (%)**				0.66^2^
No	30 (37%)	56 (40%)	86 (38.9%)	
Yes	53 (65.4%)	97 (61.4%)	188 (63.7%)	
**Child Pugh score, no. (%)**				0.5^2^
A	58 (71.6%)	106 (75.7%)	164 (74.2%)	
B	23 (28.4%)	34 (24.3%)	57 (25.8%)	
**Tumor Size**				0.51^1^
Mean ± SD	2.2 ± 0.6	2.3 ± 1.2	2.3 ± 1	
**Tumors Number, no. (%)**				0.012^1^
= 1	66 (81.5%)	96 (68%)	162 (73.3%)	
= 2	10 (12.3%)	29 (20.7%)	39 (17.6%)	
= 3	4 (4.9%)	8 (5.7%)	12 (5.4%)	
> 3	1 (1.2%)	7 (5%)	8 (3.7%)	
**BCLC Staging, no. (%)**				0.0008^2^
0	2 (2.5%)	15 (10.7%)	17 (7.7%)	
A	77 (95.1%)	105 (75%)	182 (82.4%)	
B	2 (2.5%)	20 (14.3%)	22 (10%)	
**Hyperlipidemia, no. (%)**				0.022^2^
No	55 (67.9%)	114 (81.4%)	169 (76.5%)	
Yes	26 (32.1%)	26 (18.6%)	52 (23.5%)	
**Hypothyroidism, no. (%)**				0.73^2^
No	65 (81.5%)	115 (82.1%)	180 (81.4%)	
Yes	16 (19.8%)	25 (17.9%)	41 (18.6%)	
**AFP**				0.26^1^
Mean ± SD	44.3 ± 118.9	84.7 ± 329.9	70.1 ± 273.4	
**Cholesterol (mg/dl)**				0.69^1^
Mean ± SD	152.7 ± 34.7	154.7 ± 69.9	154.1 ± 61.3	
**Triglycerides (mg/dl)**				0.3^1^
Mean ± SD	107.8 ± 46	107.2 ± 70.3	107.4 ± 63.8	
**HDL (mg/dl)**				0.91^1^
Mean ± SD	47.7 ± 18.9	48.1 ± 16.3	48 ± 17	
**LDL (mg/dl)**				0.89^1^
Mean ± SD	83.0 ± 30.8	82.4 ± 33.2	82.6 ± 32.3	
**Glucose (mg/dl)**				0.77^1^
Mean ± SD	104.7 ± 47.2	109 ± 50.4	107.9 ± 49.5	
**HB A1c**				0.3^1^
Mean ± SD	6.3 ± 1	6.9 ± 1.7	6.8 ± 1.6	
**Pre-ablation Monocytes×10**^**9**^**/L**				0.011^1^
Mean ± SD	0.6 ± 0.3	0.6 ± 0.3	0.6 ± 0.3	
**Pre-ablation Lymphocyte× 10**^**9**^**/L**				0.19^1^
Mean ± SD	1.5 ± 0.8	1.3 ± 0.7	1.4 ± 0.7	
**Post-ablation Lymphocyte× 10**^**9**^**/L**				0.83^1^
Mean ± SD	1.2 ± 0.7	1.2 ± 0.6	1.2 ± 0.6	
**Pre-ablation Neutrophils× 10**^**9**^**/L**				0.25^1^
Mean ± SD	3.4 ± 1.6	3.2 ± 1.7	3.3 ± 1.7	
**Post-ablation Neutrophils× 10**^**9**^**/L**				0.16^1^
Mean ± SD	4.5 ± 3.3	3.8 ± 2.8	4.1 ± 3	
**Pre-ablation NLR**				0.87^1^
Mean ± SD	2.9 ± 1.8	2.9 ± 1.7	2.9 ± 1.7	
**Post-ablation NLR**				0.82^1^
Mean ± SD	4.8 ± 6	4.4 ± 5.5	4.5 ± 5.7	
**Pre-ablation Platelets×10**^**9**^**/L**				0.33^1^
Mean ± SD	134.1 ± 75.4	123.3 ± 65	127.3 ± 69.1	
**Pre-ablation PLR**				0.71^1^
Mean ± SD	104.5 ± 65.2	106.1 ± 56.8	105.5 ± 59.9	
**Pre-ablation LMR**				0.69^1^
Mean ± SD	2.7 ± 1.6	2.7 ± 1.2	2.7 ± 1.4	

^1^Kruskal Wallis test, ^2^Chi-Square test, *AFP* alpha fetoprotein, *HDL* high density lipoprotein, *LDL* low density lipoprotein, *NLR* neutrophil lymphocytes ratio, *PLR* platelets lymphocytes ratio, *LMR* lymphocytes monocytes ratio

### Overall survival (OS)

**Fig. 2:**
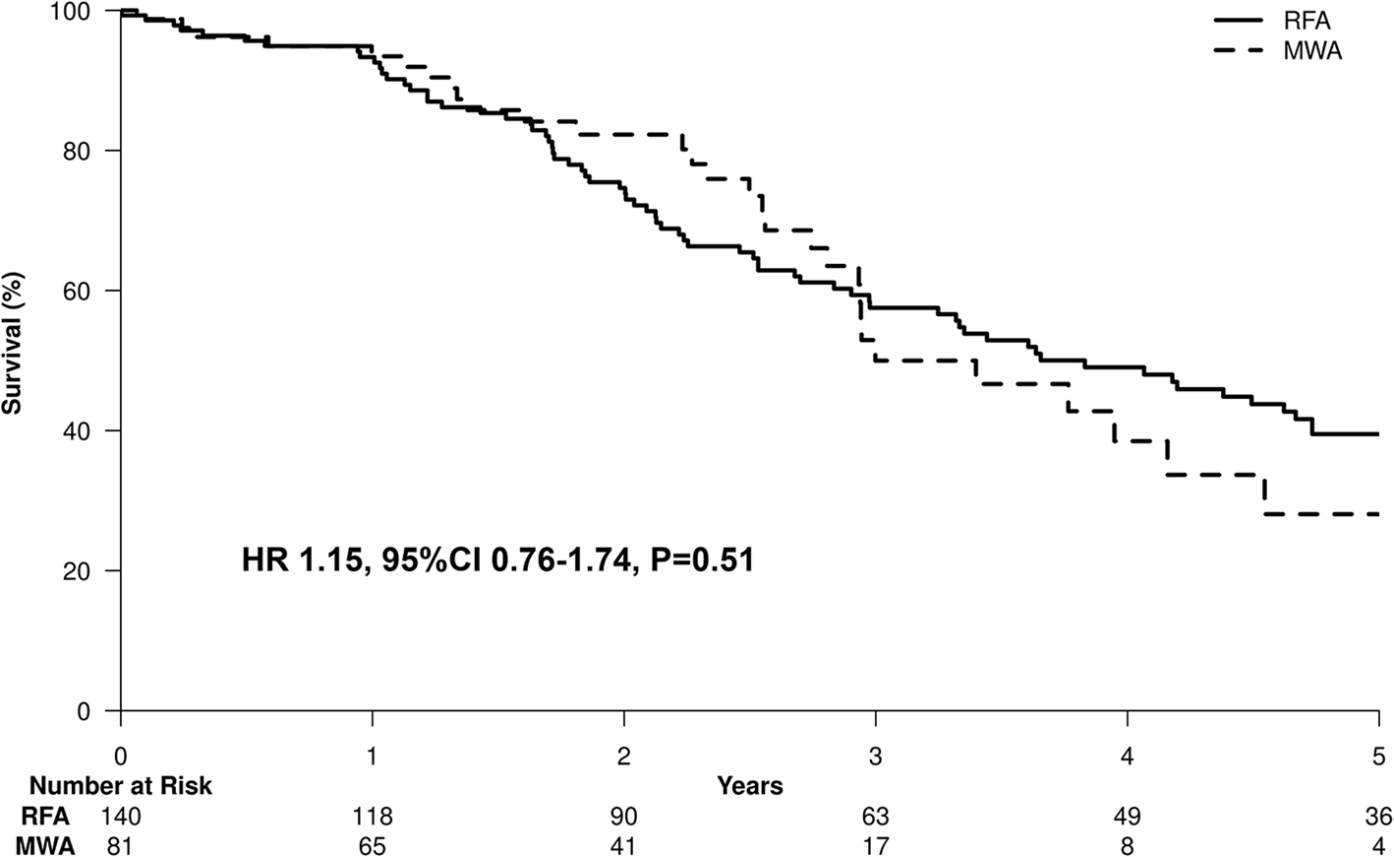
Overall survival of the study cohort by ablation type

**Fig. 3:**
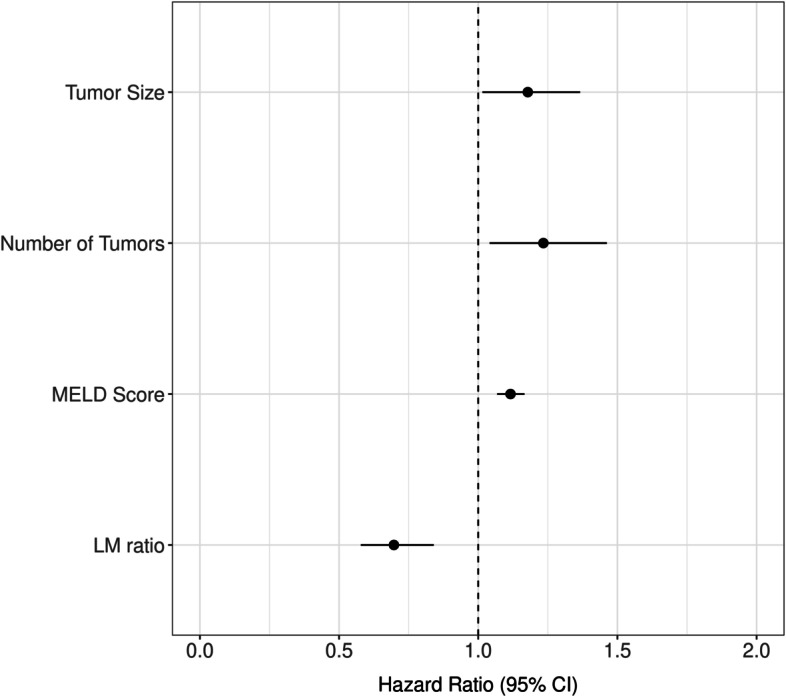
Forest plot for variables associated with overall survival

The median survival after ablation was 67.2 months. By the date of last follow up, 129 of the 221 (58.4%) patients were deceased; 83 of the 221 patients (31.2%) developed recurrence during follow up. Causes of death included HCC progression in 41 patients, liver dysfunction in 42 patients, development of other medical and surgical conditions in 20 patients, and coincidence of another cancer in 11 patients. We could not identify the cause of death in 15 patients. Of the deceased patients, 46 had no evidence of tumor recurrence. The deaths of these patients were attributable to liver dysfunction in 20 patients, other medical and surgical conditions in 12 patients, development of another cancer in 4 patients, and unknown causes in 10 patients. The 1-year, 3-year and 5-year survival rates for the RFA and MWA subgroups were 1-year: 93.5 and 93.8%, 3-year: 61.6 and 61.3% and 5-year: 46.8 and 46.1% respectively (Fig. 2). There was no significant difference in risk of death between MWA and RFA throughout the entire study and RFA and MWA had essentially equivalent Kaplan-Meier curves. Tumor recurrence was an important prognostic factor for OS by univariate Cox modeling [HR 2.31, 95% CI 1.53–3.4, *P* <  0.0001]. To explore the possible prognostic factors affecting OS, univariate and multivariate analysis of 18 variables was performed (Table 2). Variables significant in the multivariate analysis included pre-ablation lymphocyte-monocyte ratio per unit increase [HR 0.7, 95%CI 0.58–0.84, *P* = 0.0001], MELD score [HR 1.12, 95%CI 1.068–1.17, *P* <  0.0001], tumor number [HR 1.23, 95%CI 1.041–1.46, *P* = 0.015] and tumor size [HR 1.18, 95%CI 1.015–1.37, *P* = 0.031] which were independent prognostic factors for OS in HCC patients undergoing local ablation (Fig. 3).

**Table 2:** Univariate and multivariate analyses of factors determining overall survival in the entire cohort

Variable	Univariate			Multivariate		
	HR (95% CI)	overall P	*P*	HR (95% CI)	overall P	*P*
**Treatment**						
Microwave ablation	1.15 (0.76–1.74)		0.51			
Radiofrequency ablation	1.00 (Reference)					
**Age** per 10 years	1.15 (0.94–1.4)		0.17			
**Gender**						
Male	1.31 (0.91–1.9)		0.15			
Female	1.0 (Reference)					
**Cirrhosis**						
Yes	1.47 (0.72–3.02)		0.29			
No	1.00 (Reference)					
**Etiology of Liver Disease**		0.17				
HCV (positive HCV RNA)	1.38 (0.54–3.54)		0.5			
HCV (negative HCV RNA)	0.97 (0.33–2.9)		0.96			
Non-alcoholic fatty liver	1.74 (0.67–4.52)		0.25			
Alcoholic Liver disease	1.47 (0.55–3.92)		0.44			
Others	0.87 (0.33–2.28)		0.77			
HBV	1.00 (Reference)					
**Child Pugh score**						
**B**	2.39 (1.64–3.49)		< 0.001			
**A**	1.00 (Reference)					
**Portal Hypertension**						
Yes	1.61 (1.11–2.34)		0.012			
No	1.00 (Reference)					
**Tumor size per 1 unit**	1.15 (1.012–1.31)		0.032	1.18 (1.015–1.37)		0.031
**Tumor number per 1 tumor**	1.29 (1.088–1.53)		0.003	1.23 (1.041–1.46)		0.015
**BCLC Stage**		0.0009				
A	3.00 (1.31–6.86)		0.009			
B	5.80 (2.26–14.9)		< 0.001			
0	1.00 (Reference)					
**MELD Score** per point	1.08 (1.034–1.12)		< 0.001	1.12 (1.068–1.17)		< 0.0001
**Hyperlipidemia**						
Yes	1.05 (0.68–1.62)		0.83			
No	1.00 (Reference)					
**Hypothyroidism**						
Yes	1.06 (0.69–1.61)		0.8			
No	1.00 (Reference)					
**Log**^**2**^ **AFP**	1.04 (0.97–1.11)		0.32			
**Glucose (mg/dl)**	1.00 (0.99–1.005)		0.49			
**Pre-ablation NLR** per unit increase	1.17 (1.058–1.28)		0.002			
**Pre-ablation PLR**	1.03 (0.74–1.45)		0.85			
**Pre-ablation LMR** per unit increase	0.72 (0.61–0.86)		< 0.001	0.7 (0.58–0.84)		0.0001

*HCV* hepatitis C virus, *HBV* hepatitis B virus, *AFP* alpha fetoprotein, *NLR* neutrophil lymphocyte ratio, *PLR* platelets lymphocytes ratio, *LMR* lymphocyte monocyte ratio

### Disease-free survival (DFS)

**Fig. 4:**
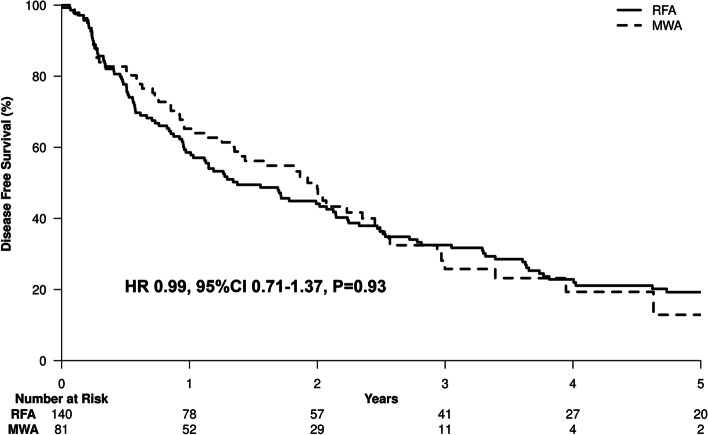
Disease-free survival of the study cohort by ablation type

After a median follow up to recurrence of 81.6 months, 130 of the 221 patients (58.8%) developed recurrent disease. Forty-seven of the 130 patients with recurrence (36.2%) were alive at the date of last follow up and 83 patients (63.8%) were deceased. We used the DFS, combining overall survival and tumor recurrence to evaluate patient outcomes given the complex overlap between the two events. The 1-year, 3-year and 5-year DFS rates of the RFA and MWA subgroups were 1 year: 58.6 and 65.3%, 3-year: 44.1 and 48.3% and 5-year: 19.3 and 12.9%, respectively (Fig. 4). On multivariate analysis, there was no statistically significant difference between the RFA and MWA treatment groups. The results were similar when limited to intra-hepatic (ablation site) recurrence rates, consistent with the majority of studies that show no statistically significant difference between RFA and MWA in terms of therapeutic efficacy, incidence of complications, and local tumor recurrence [5, 21].

**Fig. 5:**
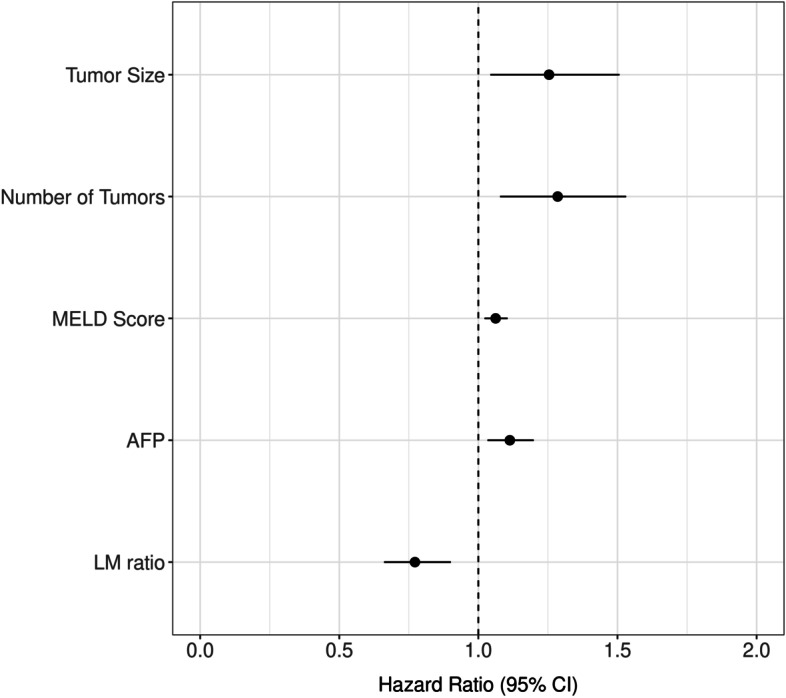
Forest plot for variables associated with disease-free survival

Univariate and multivariate analyses were performed for disease-free survival (Table 3). Pre-ablation lymphocyte monocyte ratio per unit increase [HR 0.77, 95%CI 0.66–0.90, *P* = 0.001], MELD score [HR 1.06, 95%CI 1.02–1.11, *P* = 0.002], Log^2^ AFP [HR 1.11, 95%CI 1.033–1.2, *P* = 0.005], tumor number [HR 1.29, 95%CI 1.078–1.53, *P* = 0.005] and tumor size [HR 1.25, 95%CI 1.043–1.51, *P* = 0.016] were independent prognostic factors for DFS (Fig. 5).

**Table 3:** Univariate and multivariate analyses of the factors determining disease free survival (combined death and recurrence) in the entire cohort

Variable	Univariate			Multivariate		
	HR (95% CI)	overall P	*P*	HR (95% CI)	overall P	*P*
**Treatment**						
Microwave ablation	0.99 (0.71–1.37)		0.93			
Radiofrequency ablation	1.00 (Reference)					
**Age** per 10 years	1.07 (0.91–1.26)		0.4			
**Gender**						
Male	1.32 (0.96–1.81)		0.09			
Female	1.00 (Reference)					
**Cirrhosis**						
Yes	1.24 (0.73–2.1)		0.43			
No	1.00 (Reference)					
**Etiology of Liver Disease**		0.25				
HCV (positive HCV RNA)	0.89 (0.45–1.77)		0.74			
HCV (negative HCV RNA)	0.62 (0.28–1.37)		0.23			
Non-alcoholic fatty liver	0.99 (0.49–1.98)		0.97			
Alcoholic Liver disease	0.73 (0.35–1.51)		0.39			
Others	0.60 (0.3–1.22)		0.16			
HBV	1.00 (Reference)					
**Child Pugh score**						
**B**	1.41 (1.01–1.98)		0.043			
**A**	1.00 (Reference)					
**Portal Hypertension**						
Yes	1.37 (1.004–1.87)		0.047			
No	1.00 (Reference)					
**Tumor size per 1 unit**	1.18 (1.044–1.32)		0.008	1.25 (1.043–1.51)		0.016
**Tumor number per 1 tumor**	1.31 (1.11–1.55)		0.001	1.29 (1.078–1.53)		0.005
**BCLC Stage**		0.004				
A	2.27 (1.22–4.25)		0.01			
B	3.54 (1.68–7.47)		< 0.001			
0	1.00 (Reference)					
**MELD Score** per point	1.03 (0.99–1.072)		0.085	1.06 (1.02–1.11)		0.002
**Hyperlipidemia**						
Yes	1.11 (0.79–1.57)		0.56			
No	1.00 (Reference)					
**Hypothyroidism**						
Yes	0.78 (0.53–1.16)		0.22			
No	1.00 (Reference)					
**Log**^**2**^ **AFP**	1.07 (1.005–1.14)		0.034	1.11 (1.033–1.2)		0.005
**Glucose (mg/dl)**	1.00 (0.99–1.01)		0.11			
**Pre-ablation NLR** per unit increase	1.13 (1.035–1.22)		0.006			
**Pre-ablation PLR**	0.96 (0.73–1.26)		0.77			
**Pre-ablation LMR** per unit increase	0.83 (0.73–0.95)		0.006	0.77 (0.66–0.90)		0.001

*HCV* hepatitis C virus, *HBV* hepatitis B virus, *AFP* alpha fetoprotein, *NLR* neutrophil lymphocyte ratio, *PLR* platelets lymphocytes ratio, *LMR* lymphocyte monocyte ratio

### Chronic hepatitis C in the study cohort

**Fig. 6:**
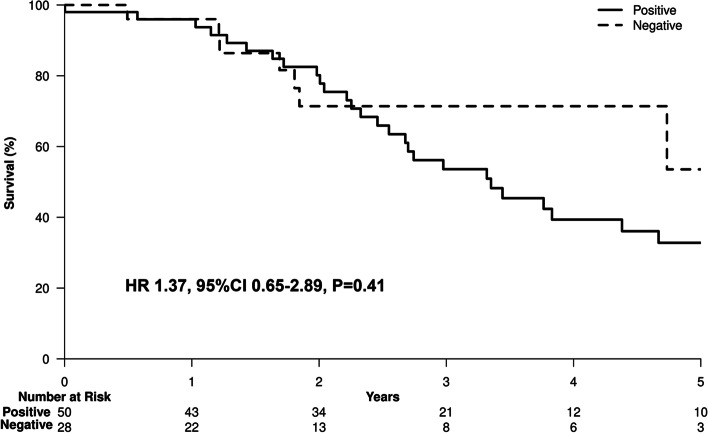
HCV RNA positive vs HCV RNA negative overall survival curves

**Fig. 7:**
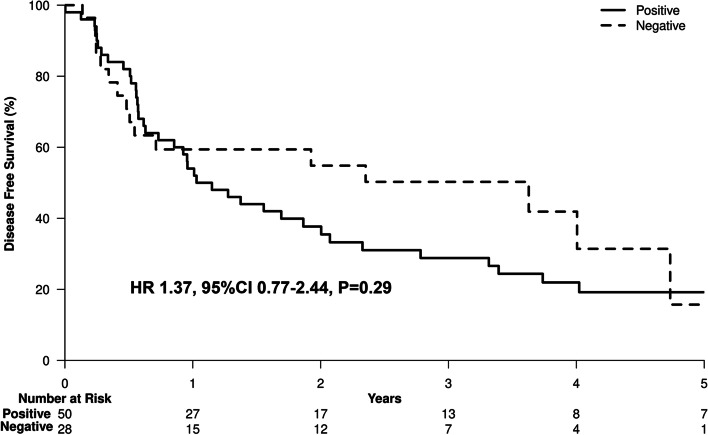
HCV RNA positive vs HCV RNA negative disease-free survival

Chronic HCV was the most common etiology of liver disease [83 of 221 patients (37.6%)]; 50 of the 221 patients (22.6%) had positive HCV RNA, 28 (12.7%) had negative HCV RNA and 5 patients had unknown HCV RNA status at the time of HCC diagnosis. Fourteen patients (6.3%) who were HCV RNA positive at HCC diagnosis received DAA after HCC treatment, while 11 patients (5%) developed HCC after cure of HCV infection. Analysis of the HCV subgroups showed that patients with positive HCV PCR had 1.4-fold higher hazard of death, with a 5-year survival rate of 32.8% compared to 53.6% in those with negative HCV PCR [HR 1.37, 95%CI 0.65–2.89, *P* = 0.41] (Fig. 6). Regarding DFS, patients with positive HCV PCR had 1.4-fold higher hazard of death and/or recurrence, with a 3-year DFS rate of 37.7% compared to 54.8% in those with negative HCV PCR, and a 5-year DFS rate of 19.2% compared to 15.7% in those with negative HCV PCR [HR 1.37, 95%CI 0.77–2.44; *P* = 0.29] (Fig. 7).

## Discussion

Progression of carcinogenesis and tumor cell metastasis depend largely on interactions occurring within the tumor microenvironment. Blood cells (endothelial cells, platelets, mast cells, lymphocytes, and macrophages), coagulation factors, stromal cells and the extracellular matrix play major roles in these interactions [22]. High neutrophil counts have been reported to be inversely correlated with survival in many solid tumor patients [23]. Neutrophils favor proliferation, invasion, and angiogenesis in cancer through enhanced synthesis and release of reactive oxygen species (ROS), causing point mutations and DNA damage [24], releasing elastase, which is proliferative, and enhancing VEGF-related angiogenesis [25]. In HCC patients a high neutrophil count has been associated with tumor progression and metastasis [26].

In contrast, enhanced infiltration of HCC tissues by T and B cells has been shown to be correlated with better patient survival [27]. Further, patients with higher intratumoral CD3+ and cytotoxic CD8+ cell infiltration were found to have longer survival [28]. CD3+ cells are considered the major immune effectors in cellular immune responses, consisting of CD4+ T helper cells and CD8+ cytotoxic T cells, which cooperate to mediate local anti-tumor immunity [29]. A suggested mechanism for the effect of lymphocytes is accelerated malignant cell apoptosis [30]. On the other hand, lymphopenia has been associated with a suppressed antitumor immune response via reduction in T4 helper lymphocytes and an increase in T8 suppressor lymphocytes [16].

Lastly, the role of monocytes as prognostic markers has been explored recently; one of the proposed underlying mechanisms is through potentiation of tumor cell escape from immune surveillance [31]. A second role is mediated via infiltration of tumor-associated macrophages (TAMs) into the HCC matrix, which has been shown to enhance tumor cell proliferation, migration, and angiogenesis [32].

Since the NLR is inexpensive and easily calculated from routine laboratory tests, a number of studies have examined the prognostic value of NLR in HCC treated using various modalities i.e., liver resection [33], transplantation [34], RFA [35], TACE [36], and sorafenib [37]. However, there is limited data about the role of the LMR in predicting prognosis of HCC patients. Yang et al. [38] reported the first study of the prognostic value of LMR in a cohort of 652 HCC patients undergoing surgical resection and reported that LMR is an independent indicator of poor OS and DFS in HCC patients treated with curative resection. Furthermore, the LMR was shown to be a better predictor of long-term survival than the NLR or PLR.

Recently, preoperative LMR of the peripheral blood of patients with HCC who underwent living donor liver transplantation was found to have utility for predicting prognosis and the LMR reflected the immune status of the HCC microenvironment. Also, high LMR was associated with poor liver function, higher expression levels of tumor markers and a higher grade of malignancy. Conversely, low LMR, both before transplant and after recurrence of HCC, was associated with poor prognosis and low LMR before transplant was identified as an independent prognostic factor, particularly among patients beyond the Milan criteria [19].

In another study from our group, we showed that LMR measured prior to initiation of sorafenib in Asian and North American cohorts with advanced HCC was statistically associated with OS. We found that on considering sorafenib treatment in those patients, a new OS nomogram incorporating LMR can aid in educating patients, prognosticating and making prognosis-based decisions for physicians [20].

Regarding patients treated with local ablation, only one study has shown an association of LMR and PLR with OS; however, the study included patients who received TACE and/or RFA, resulting in diverse baseline and tumor characteristics. In addition, essential clinical data that could potentially impact the clinical outcomes, such as the number of patients within each treatment group and the sequence of treatments, underlying liver disease, portal hypertension and BCLC stage were not available or adequately controlled for in the analysis. Finally, there were a high number of patients with portal vein thrombosis (*n* = 48 patients) vs 156 patients without portal vein thrombosis and thrombosis was not clearly indicated as malignant or due to liver dysfunction [39].

Because of the forementioned evident significance of LMR in HCC patients, we hypothesized that LMR would be a better indicator for the systemic inflammatory response than NLR or PLR in HCC patients undergoing local ablation. This study confirms the prognostic significance of the LMR, significantly influencing OS and DFS [HR 0.74, *P* = 0.0009] and [HR 0.77, *P* = 0.001] respectively. Consequently, we have demonstrated that the HCC-induced immunomodulatory effects and its key representative (LMR in this study) are of paramount significance in exploring patients’ clinical outcomes and improving treatment choice for a better patients’ prognosis.

The natural history of HCV infection ultimately culminates in progression to HCC over 20 to 40 years [40]. Viral proteins such as the HCV core protein and host immune response are major drivers of oncogenesis. Pro-oncogenic viral proteins are involved in enhanced lipogenesis, oxidative stress, and inhibition of tumor suppressor genes and cell cycle check points [41]. Immune-mediated cytokines drive chronic inflammation secondary to HCV infection, eventually leading to accumulation of mutations and subsequent malignant transformation [42].

With the wide scale use of DAA agents in treatment of HCV, a substantive body of research has focused on HCC development following DAA treatment, but less attention has been paid to co-management of active HCC and HCV [11]. In this study, we hypothesized that HCV RNA status at the time of HCC diagnosis would be of prognostic value for OS. Whether patients received DAA or cleared the virus spontaneously, patients with HCV viremia had worse 5-year survival compared to those with undetectable HCV RNA.

In this study, AFP was among the most important prognostic factors for DFS (HR 1.11; *P* = 0.005). AFP is the main serum biomarker used in surveillance, diagnosis and management of HCC. While the role of AFP in surveillance has been controversial, there is substantial evidence supporting its prognostic utility in HCC [43]. In LT patients, a high pre-operative AFP level is associated with an increased risk of tumor recurrence after transplant and inversely correlates with OS [44]. AFP has been shown to complement other prognostic markers such as the degree of tumor differentiation and microvascular spread on histopathologic examination [45]. Studies of the dynamic behavior of AFP following loco-regional therapy with transarterial chemoembolization have shown that a decrease in post-treatment AFP was associated with better OS [46]. In studies of HCC patients undergoing local ablation, it has been shown that AFP can act as a tumor associated antigen (TAA), inducing a specific CD8+ T cell response that impacts patient prognosis [47]. This is somewhat paradoxical, as an effective T cell response might be anticipated to be associated with improved outcomes, while, in contrast, high AFP levels are typically associated with poorer prognosis.

Major limitations of this study include its retrospective design, the potentially nonspecific nature of LMR and NLR as markers, since other systemic illnesses may significantly affect their values, and the fact that LMR was measured at a single time point immediately before local ablation. It is possible that measurement of multiple pre- and post-treatment values that assess dynamic changes in LMR may yield even more predictive results [38].

## Conclusion

HCC is a global health problem with progressively increasing morbidity and mortality. Local ablation is an affordable, efficient and relatively safe option for potentially curative treatment of early stage HCC and is also used as a bridge to LT. HCC induced systemic immune response represented by the LMR and underlying HCC biological behavior represented by the serum AFP may serve as novel and readily available prognostic tools in ablation patients. Prompt treatment of HCV may improve OS in those patients.

## Data Availability

The datasets used and/or analyzed during the current study are available from the corresponding author on reasonable request.

## Abbreviations

HCC: Hepatocellular carcinoma
RFA: Radiofrequency ablation
MWA: Microwave ablation
OS: Overall survival
DFS: Disease-free survival
HCV: Hepatitis C virus
PCR: Polymerase chain reaction
DAA: Direct acting antiviral
LMR: Lymphocyte Monocyte Ratio
NLR: Neutrophil Lymphocyte Ratio
PLR: Platelet to lymphocyte ratio
VEGF: Vascular endothelial growth factor
AFP: Alpha-fetoprotein
CTP: Child-Turcotte-Pugh score
MELD: Model for End-Stage Liver Disease score
BCLC: Barcelona Clinic Liver Cancer
